# Bond strength of zirconia- or polymer-based copings cemented on implant-supported titanium bases – an *in vitro* study

**DOI:** 10.1080/26415275.2021.1974301

**Published:** 2021-09-13

**Authors:** Eliann Oddbratt, Lisa Hua, Bruno R. Chrcanovic, Evaggelia Papia

**Affiliations:** a Department of Materials Science and Technology, Faculty of Odontology, Malmö University, Malmö, Sweden; b Department of Prosthodontics, Faculty of Odontology, Malmö University, Malmö, Sweden

**Keywords:** Adhesive cementation, bond strength, polymer-based materials, titanium base implant-supported dental prosthesis, zirconia

## Abstract

**Purpose:**

To evaluate the bond strength between polymer-based copings and zirconia copings as positive control, cemented on implant-supported titanium bases with different adhesive cement systems. Moreover, to evaluate if airborne-particle abrasion of polymethylmetacrylate (PMMA) would enhance the bond strength.

**Methods:**

Four groups of different materials were used to fabricate the copings, 30 in each group: airborne-particle abraded milled zirconia (TAZirconia, control group), milled PMMA (TPMMA), airborne-particle abraded milled PMMA (TAPMMA) and 3 D-printed micro filled hybrid resin (TAMFH). Each group of copings was cemented on titanium bases by three different adhesive cement systems, 10 each: Multilink Hybrid Abutment, Panavia V5, RelyX Ultimate. The specimens were stored dry at room temperature for 24 h, subjected to thermocycling for 5000 cycles followed by evaluating the bond strength by tensile strength test.

**Results:**

TPMMA and TAPMMA cemented with Multilink Hybrid Abutment showed statistically significant lower bond strength in comparison to TAZirconia and TAMFH. No difference was observed between the latter two. TPMMA, TAPMMA and TAMFH had a statistically significant lower bond strength compared to the control group when cemented with Panavia V5. TPMMA and TAPMMA cemented with Rely X Ultimate showed statistically significant lower bond strength in comparison to the control group.

**Conclusion:**

Almost all experimental groups, except 3 D-printed MFH, performed inferior than the positive control group where the highest bond strength was reported for the cementation of zirconia copings cemented with Panavia V5 or Rely X Ultimate. Airborne-particle abrasion did not improve the bond strength of the PMMA, except when Multilink Hybrid Abutment was used.

## Introduction

Replacing missing teeth using dental implants has become a well-established approach in oral rehabilitation, and implant-supported fixed dental prostheses have become one of the most common prosthodontic restorations of today [1]. Implant-supported fixed dental prostheses can be either screw-retained, cement-retained, or a combination of both, and each method has their advantages and disadvantages [2].

When it comes to cement-retained restorations, although improved mechanical properties are important for the long-term performance of an oral restorative material, the clinical success of fixed prostheses seems to be strongly dependent on the cementation procedure [3]. A strong bond between the restoration material, the cement and the base are essential for the longevity of the restoration [4]. Factors that are important for the retention of cemented restorations, include the cement type, cement gap, geometry, height, surface area, and roughness of the abutment [5,6]. Regardless of abutment height and geometry, the bond strength between the luting agent and the bonding surfaces is determined by the strength of the chemical bonds, mechanical interlocking, and surface roughness [7].

The combination of CAD/CAM technology and digital workflows has provided new treatment and planning strategies, better material quality through standardization of the manufacturing process, and reproducibility. Manufacturing processes supported by CAD/CAM technology have enabled subtractive (milling) or additive (3 D-printing) procedures. Polymer-based materials have attracted an increasing interest due to their biological and mechanical properties, their ease in processing and lower costs in production, which allows them to be applied for a wide range of applications [8]. Polymer-based materials are often used for the fabrication of temporary restorations to restore function and esthetics during the osseointegration of dental implants or during the final restoration process. Temporary restorations can be fabricated from composite, polyetheretherketone (PEEK) and polymethylmetacrylate (PMMA) based materials [9]. In the daily clinical practice, there are many different dental materials used for abutments and since the polymer-based materials are relatively new, laboratory and clinical studies are highly needed. Therefore, there is an interest to evaluate the bond strength between these materials for temporary restorations and establish a cement protocol for short- or long-term use and eventually have to be removed. In the clinic practice, tooth-supported restorations with CAD/CAM systems combined with adhesive cementation are frequently used and well documented, identifying the challenge of accomplishing a stable bond to zirconia-based restorations. However, cementation protocols for titanium-bases have been scarcely explored. Thus, it is essential to have knowledge of adhesive systems and the cement protocol for temporary and permanent restorations [10]. The fact that temporary restorations are expected to be *in situ* for a shorter period of time in comparison to permanent restorations, do not make them less important. A well-designed temporary restoration with a stable bond can protect the prepared tooth, provide better healing of the surrounding periodontal tissues, and reduce the number of appointments, as decementation of temporary restorations cause increased chair-side time with additional appointments. The present study was designed to evaluate a material for permanent restorations, that is, zirconia and materials for temporary restorations, that is, polymer-based materials which needs to be further assessed for clinical recommendations.

As the longevity of cement-retained implant restorations is subjected to the influence of several factors, also facing the fact that manufacturers have developed several materials for restorations and several bonding agents including different adhesive monomers, the aim of this *in vitro* study was to evaluate the bond strength between polymer-based copings with zirconia copings as positive control, which are cemented on implant-supported titanium bases with different adhesive cement systems. Moreover, to evaluate if pre-treatment with airborne-particle abrasion of PMMA restoration, according to the material manufacturer recommendation, would enhance the bond strength. The null hypotheses were that the mean tensile bond strength value between the coping and the titanium base will not be affected by the restoration material and the adhesive cement system used. Furthermore the bond strength between PMMA copings and the titanium base will not be affected by the pre-treatment with airborne-particle abrasion.

## Materials and methods

### Coping fabrication

**Figure 1.**
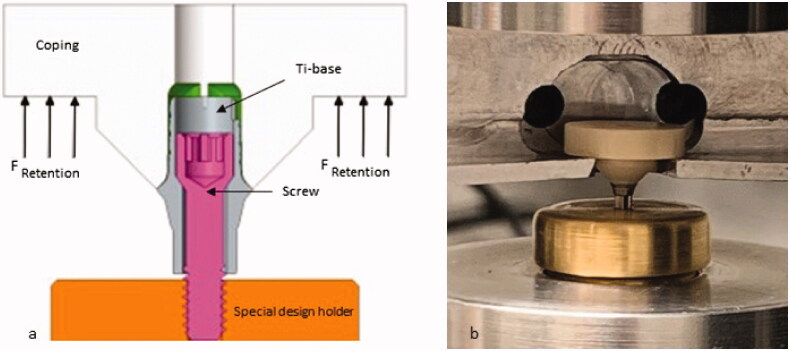
(a) Schematic figure of specimen (b) test set up.

One hundred and twenty titanium bases (Elos Accurate® Hybrid Base™ Engaging, Elos MedtechEngvej 33, Gørløse, Denmark) with an abutment height of 3 mm and a cementation area of 24.74 mm^2^ were used. The titanium bases were screwed on to an implant analog with titanium abutment screws. Four groups of different materials were used to fabricate the copings to fit the titanium bases. A total of 30 zirconia copings were milled from IPS e.max ZirCAD LT, translucent 3Y-TZP, (Ivoclar Vivadent AG, Schaan, Liechtenstein) and 60 PMMA copings were milled from Telio CAD blocks (Ivoclar Vivadent AG, Schaan, Liechtenstein) using a CAD/CAM milling machine (Datron D5, CAM Hyper dent 8.0, DATRON AG, Mühltal Germany). The group of zirconia copings were subjected to airborne-particle abrasion and was considered as the control group (TAZirconia). The 60 PMMA copings were divided into two groups of 30 copings each, the PMMA group not subjected to airborne-particle abrasion (TPMMA) and the PMMA group subjected to airborne-particle abrasion (TAPMMA). The fourth group consisted of 30 copings 3 D-printed with Micro Filled Hybrid material (TAMFH) (Micro Filled Hybrid C&B, Nextdent B.V., Soesterberg, Netherlands), using a 3 D-printing machine (Nextdent 5100 3 D sprint, Nextdent B.V., Soesterberg, the Netherlands) and subjected to airborne-particle abrasion. Each specimen of the study consisted of a titanium-base-cement-coping set (Figure 1(a)).

### Pretreatment

Copings from the groups TAZirconia, TAPMMA and TAMFH were subjected to airborne-particle abrasion with a pressure of 1 bar with aluminum oxide (Al_2_O_3_, mean particle size of 110 µm, Alox Cobra 110 µm, Renfert, Hilzingen, Germany) for 5 s, with the distance between the airborne-particle abrasion nozzle kept with a 90 degree of angulation and 10 mm away from the copings bonding surface. All copings were cleaned in an ultrasonic bath in water for 2 min and dried with oil-free air, after which the bonding surfaces were cleaned with ethanol with a disposable brush.

### Coping cementation to the titanium base

**Figure 2.**
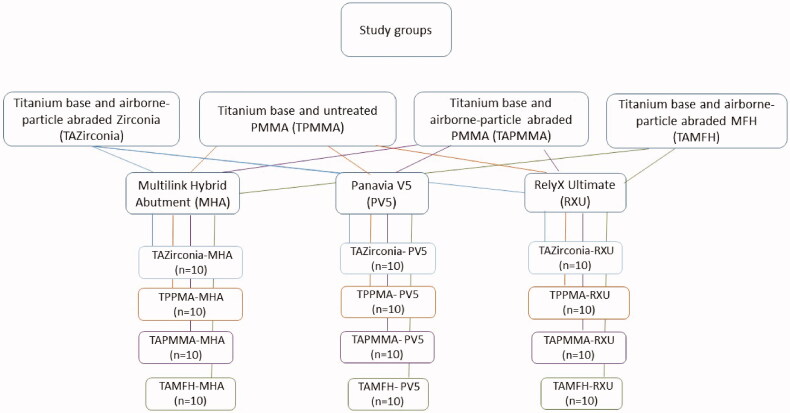
The study groups including abbreviation and subgroups with the different cements.

All copings were cemented to the titanium bases immediately after the cleaning process in order to minimize surface contamination. The copings were cemented to the titanium bases strictly following the manufacturer’s instructions. Three usual resin cements were used, namely Multilink Hybrid Abutment (Ivoclar Vivadent AG, Schaan, Liechtenstein), Panavia V5 (Kuraray Noritake, Tokyo, Japan), and RelyX Ultimate (3 M ESPE, Seefeld, Germany), Table 1 and Figure 2. Two dental implant analogs (Elos Accurate^®^ Model Analog, Elos Medtech Engvej 33, Gørløse, Denmark) were aligned vertically in the center of a plastic ring. Cold polymerizing acrylic resin (Meliodent, Heraues Kulzer GmbH, Hanau, Germany) was poured and allowed to initially set, and then placed in a pressure polymerization unit (Palamat elite, Kulzer GmbH, Hanau, Germany) for 10 min at 55 °C with a pressure of 2 bar. The implant analogs were used to fixate the base and the coping during the cementation procedure.

**Table 1.** The included adhesive cement systems, type and main components.

Cement	Type/Curing	Main composition
Multilink Hybrid Abutment (MHA) including SR Connect	Resin-based cement/ Auto polymerization	Components: Dimethacrylate, HEMA, fillers (barium glass, ytterbium trifluoride, spheroid mixed oxides, titanium dioxide) Components: Methylmethacrylate, polymethylmethacrylate, dimethacrylates, initiators
Panavia V 5 (PV5) including Clearfil Ceramic Primer Plus	Resin-based cement/ Dual polymerization	Components: Bisphenol A- Bis-GMA, diglycidyl methacrylate triethyleneglycol dimethacrylate, silanated barium glass filler, silanated fluoroaluminosilicate glass filler colloidal silica, aluminum oxide filler, hydrophobic aromatic dimethacrylate, TEGDMA, hydrophilic aliphatic dimethacrylate dl-Camphorquinone, initiators accelerators, pigments Components: Original MDP adhesive monomer, y-MPS silane monomer
RelyX Ultimate (RXU) including Scotchbond Univeral Adhesive Primer	Resin-based cement/ Dual polymerization	Components: Methacrylate monomers, methacrylate monomers radiopaque, silanated fillers radiopaque alkaline (basic) fillers, initiator components, stabilizers, rheological additives, pigments, fluorescence dye Components: MDP phosphate monomer dimethacrylate resins HEMA Vitrebond™ copolymer, filler, ethanol, water, initiators, silane

Cementation with Multilink Hybrid Abutment (MHA). A coupling agent Monobond Plus (Ivoclar Vivadent AG, Schaan, Liechtenstein) was applied to the titanium bases bonding surface for 60 s and dried with oil-free air. TAZirconia copings were treated with the same coupling agent for 60 s and dried with oil-free air. In the TAMFH, TPMMA and TAPMMA groups, a thin layer of SR Connect (Ivoclar Vivadent AG, Schaan, Liechtenstein) was applied on the bonding surface with a disposable brush for 30 s. Subsequently, the surfaces were polymerized with a polymerizing light (Translux Power Blue, Heraeus Kulzer, Milan, Italy) for 10 s each of the four sides. A thin cement layer was applied with a syringe to the surface of the titanium bases and to the surface of the copings. The titanium bases and copings were initially seated with finger pressure, then a 15 N load was directed axially onto the cemented specimen. Any excess of cement remaining at the margin was removed using a disposable brush. Glycerin gel (Liquid Strip glycerin gel, Ivoclar Vivadent AG, Schaan, Liechtenstein) was applied on the margin to prevent the formation of an inhibition layer. The gel was left on the margin until the polymerization was completed, by letting it polymerize chemically for 7 min after placement under a constant pressure of 15 N and subsequently the glycerin gel was removed by water rinsing.

Cementation with Panavia V5 (PV5). A coupling agent, Clearfil Ceramic Primer PLUS (Kuraray Noritake, Tokyo, Japan) was applied to the bonding surface of the copings of all material groups and to the bonding surface of the titanium bases with an applicator brush tip. After the application, the entire bonding surface was carefully dried for 5 s using oil-free air flow for 5 s. With a syringe, the mixed cement paste was applied over the entire bonding surface of the copings. The titanium bases and copings were cemented together according to the same cementation process described for MHA. Any excess of cement remaining at the margin was removed using a disposable brush followed by light-polymerization using the dental polymerizing unit (Translux Power Blue, Heraeus Kulzer, Milan, Italy) for 3–5 s on each of the four sides. The cement was allowed to further polymerize chemically by letting it set for 10 min after placement under a constant pressure of 15 N, according to the cementation process described for MHA.

Cementation with RelyX Ultimate (RXU). The coupling agent Scotchbond Universal Adhesive (3M ESPE, Seefeld, Germany) was applied to the bonding surface of the copings and the titanium bases for 20 s, and then carefully dried using oil-free air flow for 5 s. The margins were light-polymerized using the dental polymerizing unit (Translux Power Blue, Heraeus Kulzer, Milan, Italy) for 20 s on each of the four sides. The cement was allowed to polymerize chemically by letting it set for 6 min after placement, under a constant pressure of 15 N, according to the cementation process described for MHA.

In total, there were 12 groups of 10 specimens each, with four different materials and three different adhesive cements Figure 2.

### Thermocycling

After cementation, all specimens were stored dry at room temperature (20 ± 1 °C) for 24 h and subsequently subjected to thermocycling (Termocycler THE-1100, SD Mechatronik, Feldkirchen-Westerham, Germany) for 5000 cycles before the tensile strength test. One cycle period lasted for a total of 60 s, of which 20 s in respective two baths, with temperatures of 5 °C and 55 °C and with 10 s transport time between the baths.

### Tensile strength test

The test setup was designed in a way that each specimen was screwed into a specially made brass metal holder with a torque force of 7.5 N (Figure 1(b)). The bond strength was measured by a universal testing machine (Instron Universal Machine, Instron Co., Norwood, USA) with a crosshead speed of 0.5 mm/min [11]. Each specimen was mounted in the testing machine using a custom-made jig. The load at failure was measured in Newton (N) and also converted to Megapascal (MPa) by dividing N with the area of the titanium base.

### Failure analysis

The surfaces of the titanium bases were analyzed where the fractures were categorized into adhesive or mixed fractures. A fracture was classified as an adhesive fracture when most of the remnant of the cement was left on either the coping, (intaglio surface) or the titanium surface. For mixed fractures, both cohesive and adhesive fractures were required on both surfaces. The assessments were performed using a microscope (Wild M3, M7A Wild Heerbrugg, Heerbrugg, Switzerland) with 10 x magnification.

### Statistical analysis

A descriptive analysis was performed based on mean, standard deviation (SD), and percentage values. Kruskal–Wallis test with *post hoc* Dunn test with Bonferroni adjustment was used to compare the bond strength mean values between the different groups. The level of statistical significance was set at *p* < .05. All data were analyzed using IBM SPSS Statistics for Windows, version 26 (IBM Corp., Armonk, NY, USA).

## Results

### Bond strength test

The results are summarized and presented in Table 2 that shows detailed results for the 12 groups. The following findings were observed, according to the cemented used.

**Table 2.** Bond strength results of the 12 groups, by material and cement used (values in Newton and MPa).

Material	Cement	Mean ± SD (min, max) Newton (upper values) MPa (lower values)	Median (interquartile range 25%, 75%)	Groups a - l for statistical analysis	*p* < .05^a^
	MHA	147 ± 37 (94, 193)	143 (111, 193)	a	b–i, k, l
		5.9 ± 1.5 (3.8, 7.8)	5.8 (4.5, 7.8)		
TAZirconia	PV5	229 ± 26 (202, 278)	221 (206, 254)	b	a, d–k
		9.3 ± 1.1 (8.2, 11.2)	8.9 (8.3, 10.3)		
	RXU	226 ± 30 (176, 268)	229 (202, 248)	c	a, d–k
		9.1 ± 1.2 (7.1, 10.8)	9.3 (8.2, 10.0)		
	MHA	32 ± 28 (1, 74)	20 (7, 61)	d	a–c, j–l
		1.3 ± 1.1 (0.04, 3.0)	0.8 (0.3, 2.5)		
TPMMA	PV5	27 ± 15 (2, 53)	27 (19, 39)	e	a–c, j–l
		1.1 ± 0.6 (0.08, 2.1)	1.1 (0.8, 1.6)		
	RXU	38 ± 23 (15, 80)	30 (18, 57)	f	a–c, j–l
		1.5 ± 0.9 (0.6, 3.2)	1.2 (0.7, 2.3)		
	MHA	48 ± 15 (28, 81)	48 (37, 54)	g	a–c, j–l
		1.9 ± 0.6 (1.1, 3.3)	1.9 (1.5, 2.2)		
TAPMMA	PV5	37 ± 12 (25, 61)	34 (26, 46)	h	a–c, j–l
		1.5 ± 0.5 (1.0, 2.5)	1.4 (1.1, 1.9)		
	RXU	37 ± 22 (17, 77)	28 (20, 55)	i	a–c, j–l
		1.5 ± 0.9 (0.7, 3.1)	1.1 (0.8, 2.2)		
	MHA	146 ± 63 (35, 221)	155 (89, 202)	j	b–i, k, l
		5.9 ± 2.5 (1.4, 8.9)	6.3 (3.6, 8.2)		
TAMFH	PV5	105 ± 37 (48, 176)	107 (77, 127)	k	a–j, l
		4.2 ± 1.5 (1.9, 7.1)	4.3 (3.1, 5.1)		
	RXU	221 ± 33 (191, 299)	208 (202, 235)	l	a, d–k
		8.9 ± 1.3 (7.7, 12.1)	8.4 (8.2, 9.5)		

Cement systems: MHA: Multilink Hybrid Abutment; PV5: Panavia V5; RXU: RelyX Ultimate. SD: standard deviation; min: minimum; max: maximum.

^a^The letters shown in this column indicate the groups that present mean values statistically significant different from the group presented in the row of the Table, specified under the column ‘Group’.

For the groups cemented with MHA, TPMMA and TAPMMA showed statistically significant lower bond strengths in comparison to TAZirconia (*p* <.001 and *p* < .001, respectively) and TAMFH (*p* < .001 and *p* = .001, respectively). No difference was observed between TAMFH and TAZirconia (*p* = .853). The groups that were cemented with PV5 showed that TAZirconia showed significantly higher bond strength compared with the experimental groups (TPMMA: *p* < .001, TAPMMA: *p* <.001, TAMFH: *p* < .001). For the groups cemented with RXU, the groups TPMMA and TAPMMA showed statistically significant lower bond strengths in comparison to TAZirconia (*p* < .001 and *p* < .001, respectively). No difference was observed between TAMFH and TAZirconia (*p* = .481).

After thermocycling, it was possible to see cracks in the TAMFH copings; however, the TAMFH groups showed higher bond strength in comparison to TPMMA (for MHA: *p* < .001, for PV5: *p* < .001, for RXU: *p* < .001, respectively) and TAPMMA (for MHA: *p* = .001, for PV5: *p* < .001, for RXU: *p* < .001, respectively), regardless of the cement used. MHA had a statistically significance lower mean value in bond strength than PV5 and RXU for the TAZirconia group (*p* < .001 and *p* < .001, respectively). There was no difference in the mean bond strength between airborne-particle abraded or not abraded PMMA (for MHA: *p* = .280, for PV5: *p* = .165, for RXU: *p* = .912, respectively). TAMFH cemented with RXU was the only group that showed comparable mean value in bond strength to TAZirconia group (*p* = .481).

### Failure analysis

Adhesive dislodgement of the cement between the coping and titanium abutment was present in all groups after the tensile strength test. For the MHA cement system, the remnant of the cement was left on the coping, irrespectively material of the coping and for the other cement systems, PV5 and RXU, the remnant of the cement was left on the titanium surface. No fractures occurred in the copings nor titanium bases.

## Discussion

The results of the present studies suggest that the type of resin cement and the coping material regardless airborne-particle abrasion have different bond strength between the copings to the titanium bases, and therefore, the hypotheses are rejected.

The adhesive cement systems used in the present study have an indication for permanent use in clinical practice. The results from the bond strength test showed that the cement system Multilink Hybrid Abutment (MHA) had significant lower value than Panavia V5 (PV5) and Rely X Ultimate (RXU) in general. The bond strength of the adhesive cement system depends on several factors such as micro- and macromechanical retention, chemical retention, dental materials used, type of adhesive resin cement and how the operator handle the materials in the dental laboratory, that is, if the instructions are followed according to the manufacturer [12]. The cement composition influences the bond strength and the adhesive resin cement systems tested contain different filler particles and matrix. The majority of studies reports that the highest retention was achieved when phosphate monomers [13], and more specific 10-methacryloyloxy-decyl dihydrogenphosphate (MDP) were present in the cements’ composition [14,15]. However, MHA is polymerized through auto polymerization which differs from PV5 and RXU, which both are dual polymerized and have been reported to obtain the high bond strength even after thermocycling [16].

The TAZirconia group was the one with the general highest values in bond strength in comparison to the other groups of materials. Something that may help to explain this finding is the fact that zirconia can react with phosphate ester monomers, which is why MDP-containing primers or resin cements can improve zirconia resin cement bonding [14–18]. Airborne-particle abrasion is known to form surface roughness and irregularities and to increase the surface area and wettability, thus allowing resin cement to flow into the surface [19]. However, no significant differences in bond strength were observed between the two PMMA groups (TAPMMA and TPMMA). This may be due to the fact that some of the alumina particles can stay inside the material and reduce the bonding between the titanium base and the coping [7]. Moreover, airborne-particle abrasion of PMMA may have not significantly increased the roughness and irregularities of its surface, regardless of the cement used, although the roughness of the materials surface used in the present study was not verified. Pretreating the titanium-base with airborne-particle abrasion was excluded in this study, due to the recommendation of the manufacturer, but also that the pretreatment can affect the cement gap, causing too thick cement layer and inducing stress within the cement. Studies have report that the bonding between restoration and titanium abutment can be increased when the titanium surface or the cementation surface of the restoration are subjected to airborne-particle abrasion [20,21]. However, there are contradicted results presented as well, concluding that the bond strength is reduced after aluminum oxide abrasion [7,19] or it does not have any impact on the bonding between cement and restoration [22], all depending on the particle size, pressure and distance of subjected airborne particle abrasion. The type, shape and surface including treatment such as airborne-particle abrasion before cementation of the titanium abutment has an impact on the bonding between restoration and abutment as well [20,21]. Other factors that negatively affect the binding is the thermocycling procedure [23,24]. The mechanical properties of resin materials are affected by the manufacturing process [25]. After thermocycling, it was possible to see superficial cracks in the TAMFH copings, which may occur in polymer-based materials due to the stress obtained due to the temperature changes [23,24]. The materials used for 3 D printing is still under development and more research is needed to fully understand the properties when different stress loads are applied. Even if there were cracks in the specimens of the TAMFH group, this may have not influenced the bond strength of the cements, as the mean values for the TAMFH group were higher than the PMMA groups, suggesting that, regardless of the cement system used, bonding was better to the 3 D-printed polymeric material. It is difficult to draw a conclusion if the bond strength is sufficient for clinical use for polymer-based restorations bonded to titanium bases, due to the fact that the material is for temporary restorations under the healing process.

One of the limitations of the present work in the fact that only thermocycling was performed, not mechanical aging. Thermo-mechanical aging, in order to simulate chewing, would have a deleterious effect on the bonded interface, and the results could potentially be different. Furthermore, the tensile test used includes a device were the load is applied perpendicular to the specimen, but does not include the total circumference. The stress distribution can affect the coping by having unequal stress, creating a bending force of the coping and the bonded interface, however, none of the copings from all groups showed any fractures after the tensile bond strength test. Moreover, no evaluation of the marginal gap between the titanium base and the copings was performed after cementation and after thermocycling, furthermore the combination of thermo-mechanical aging may have an impact on the bonding interface which may alter the pull-out forces [26].

## Conclusions

Within the limitations of this *in vitro* study, the following conclusions were drawn: All experimental groups performed inferior than the positive control group where the highest bond strength was reported for the cementation of zirconia copings when dual polymerized cement systems were used. The 3 D-printed polymer-based material had higher bond strength regardless of cement system used in comparison to PMMA. Airborne-particle abrasion did not improve the bond strength of the milled PMMA copings to the titanium base in comparison to PMMA copings not subjected to airborne-particle abrasion for the tested cement systems except when using Multilink Hybrid Abutment.
